# An Optically Transparent Metamaterial Window Based on Metal Meshes With Integrated Absorption and Transmission

**DOI:** 10.1002/nap2.70265

**Published:** 2026-08-23

**Authors:** Chaoyue Li, Chenyang Cui, Wangqi Mao, Pingping Min, Tingting Yu, Yang Zhang, Yaqiang Zhang, Chi Zhang, Hongxing Dong, Long Zhang

**Affiliations:** ^1^ Hangzhou Institute for Advanced Study, University of Chinese Academy of Sciences Hangzhou China; ^2^ Harbin Institute of Technology Harbin China; ^3^ Zhengzhou Advanced Research Institute of Harbin Institute of Technology Zhengzhou China; ^4^ Key Laboratory of Materials for High‐Power Laser Shanghai Institute of Optics and Fine Mechanics, Chinese Academy of Sciences Shanghai China; ^5^ Hangzhou Hengying Technology Co., Ltd Hangzhou China; ^6^ Hangzhou Institute of Optics and Fine Mechanics Hangzhou China

**Keywords:** metasurface, microwave absorption, optical transparency, transmission window

## Abstract

This work presents an absorptive–transmissive integrated metamaterial window using a quartz substrate and micrometer‐scale metallic meshes. The structure employs a three‐layer design comprising a resonant layer, a dielectric spacer, and a frequency‐selective surface. It achieves an average visible transmittance of 68.5% over 380–780 nm, along with a dual‐band microwave response: an average absorption of 85% in the 5–11.4 GHz band and a transmittance that rises from 20 GHz and peaks around 34 GHz. The low‐frequency absorption originates from localized surface plasmon resonance in the top layer, whereas the high‐frequency transmission results from multilayer interference. This work provides a design strategy for integrated windows capable of simultaneously managing optical and microwave radiation.

## Graphical abstract

We demonstrate an optically transparent metallic‐mesh electromagnetic window that integrates radar‐frequency absorption, millimeter‐wave transmission, and visible‐light transparency within a single passive architecture, providing a new approach for multifunctional electromagnetic control.

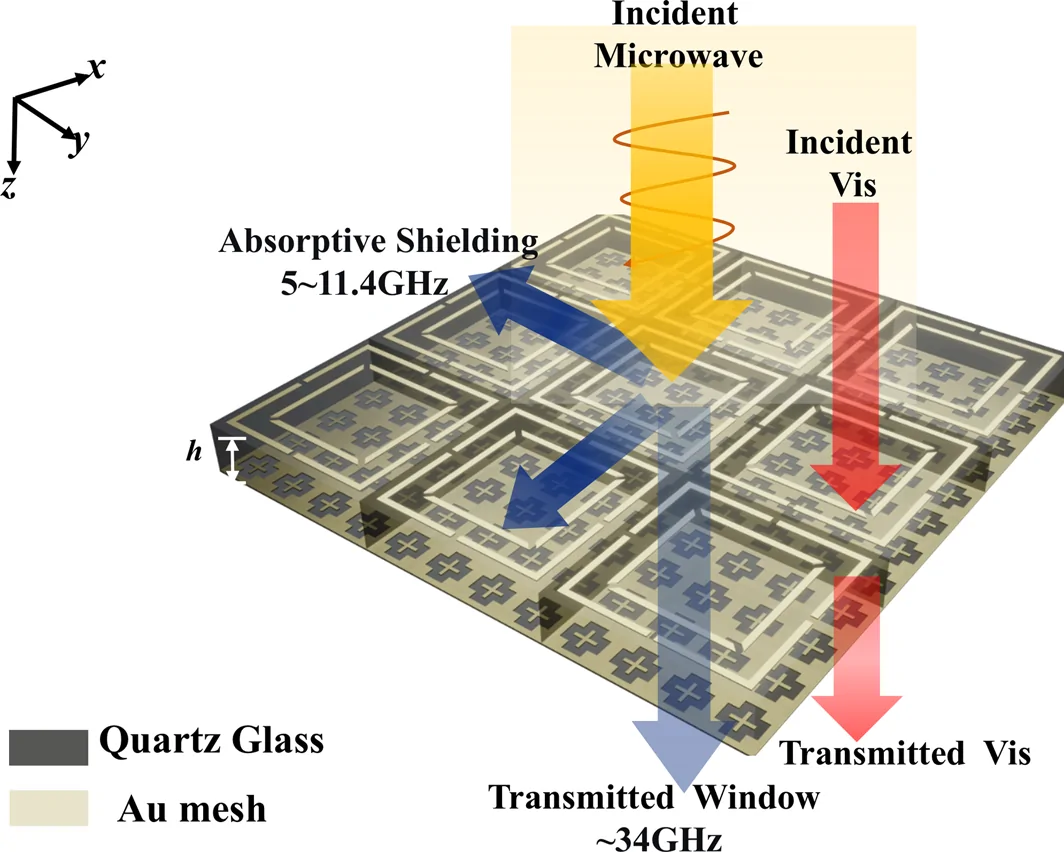

## 1 Introduction

Modern integrated electronic systems, particularly those deployed on aerospace and special vehicle platforms, increasingly demand multifunctional electromagnetic windows [1, 2, 3, 4, 5, 6, 7]. Such a window must integrate multiple critical functions within a single aperture: effective broadband absorption in radar bands such as C and X for stealth [8, 9, 10], high transmission in communication bands such as Ka for unimpeded data flow [11, 12, 13], and high visible‐light transmittance for optical vision [14, 15]. However, these optical and electromagnetic requirements are inherently coupled, making the simultaneous realization of broadband absorption, frequency‐selective transmission, and high optical transparency a challenging design problem rather than a simple combination of individual functional layers [3, 16, 17, 18, 19, 20, 21, 22, 23].

To address this design challenge, frequency‐selective surfaces (FSS) and metamaterials have been extensively studied for their spatial filtering and wave‐manipulation capabilities [22, 24, 25, 26, 27, 28, 29]. Based on the established FSS theory and design methodology, frequency‐selective structures have been extended from conventional transmission/reflection control toward electromagnetic absorption, such as the wideband absorbing frequency‐selective radome proposed by Costa and Monorchio [30, 31]. Subsequently, Zhang et al. developed an FSS‐based dual‐band broadband absorber, whereas Mao et al. and Geng et al. further extended these concepts toward frequency‐selective rasorbers and multifunctional stealth metasurfaces with simultaneous absorption and transmission control [32, 33, 34]. For transparent electromagnetic windows, metallic meshes and other transparent structures have been explored to integrate optical transparency with microwave manipulation. Zhang et al. demonstrated a visible–near‐infrared transparent frequency‐selective metasurface based on metallic meshes, whereas Shen et al. and Wang et al. reported transparent absorbers using water‐substrate and double‐layer mesh configurations, respectively [35, 36, 37]. Chen et al. further demonstrated a transparent and broadband diffusion metasurface using metallic meshes, achieving high optical transparency together with effective electromagnetic shielding performance [38]. Compared with conventional transparent electromagnetic architectures, micrometer‐scale metallic meshes provide high conductivity, low optical filling ratio, and substantial geometric freedom, offering a promising platform for simultaneously engineering optical and microwave functionalities [39, 40, 41, 42, 43]. However, existing metallic‐mesh‐based electromagnetic windows are generally designed for either frequency‐selective transmission or broadband absorption, leaving the simultaneous realization of absorption and transmission functionalities within a compact metallic‐mesh architecture insufficiently explored. Such an absorptive–transmissive design is highly desirable for simultaneously enabling stealth and communication functions while minimizing the thickness, interface losses, and optical degradation inherent to multilayer structures.

Building upon this foundation, this paper proposes and experimentally demonstrates an absorptive–transmissive integrated metamaterial window based on a rigid quartz‐glass substrate and micrometer‐scale metallic meshes. By combining the mechanical robustness of a rigid substrate with the high conductivity and structural tunability of metallic meshes, the proposed platform enables the co‐optimization of microwave absorption, frequency‐selective transmission, and visible‐light transparency within a single passive architecture. Specifically, the top resonant layer and bottom transmission FSS are simultaneously engineered through a coordinated topology design, achieving multifunctional electromagnetic responses without introducing additional functional layers or active components. The device demonstrates an average broadband absorption of 85% within the 5–11.4 GHz band, forms a selective transmission window around 34 GHz, and maintains an average transmittance of 68.5% across the 380–780 nm visible spectrum. A full‐chain verification is completed through simulation‐based optimization, fabrication via standard photolithography, and free‐space measurement. Furthermore, the underlying physical mechanisms are elucidated via electromagnetic field analysis and a multiple‐reflection interference model: low‐frequency absorption originates from localized surface plasmon resonance (LSPR) and ohmic loss, whereas high‐frequency transmission is governed by coupling interference within a Fabry–Pérot cavity. This study establishes a topology‐driven design strategy for transparent multifunctional electromagnetic windows requiring broadband microwave absorption, frequency‐selective transmission, and high optical transparency.

## 2 Design and Methodology for Absorptive–Transmissive Integration

### 2.1 Metasurface Design

The design evolution of the proposed metamaterial window is illustrated in Figure 1. Full‐wave simulations and parametric optimizations were performed using CST Microwave Studio. Periodic boundary conditions were applied to simulate an infinite array under normal incidence. The optimized structure consists of a resonating top layer, a dielectric spacer, and a bottom frequency‐selective surface (FSS), forming a three‐layer metallic‐mesh architecture on a rigid quartz substrate.

**FIGURE 1:**
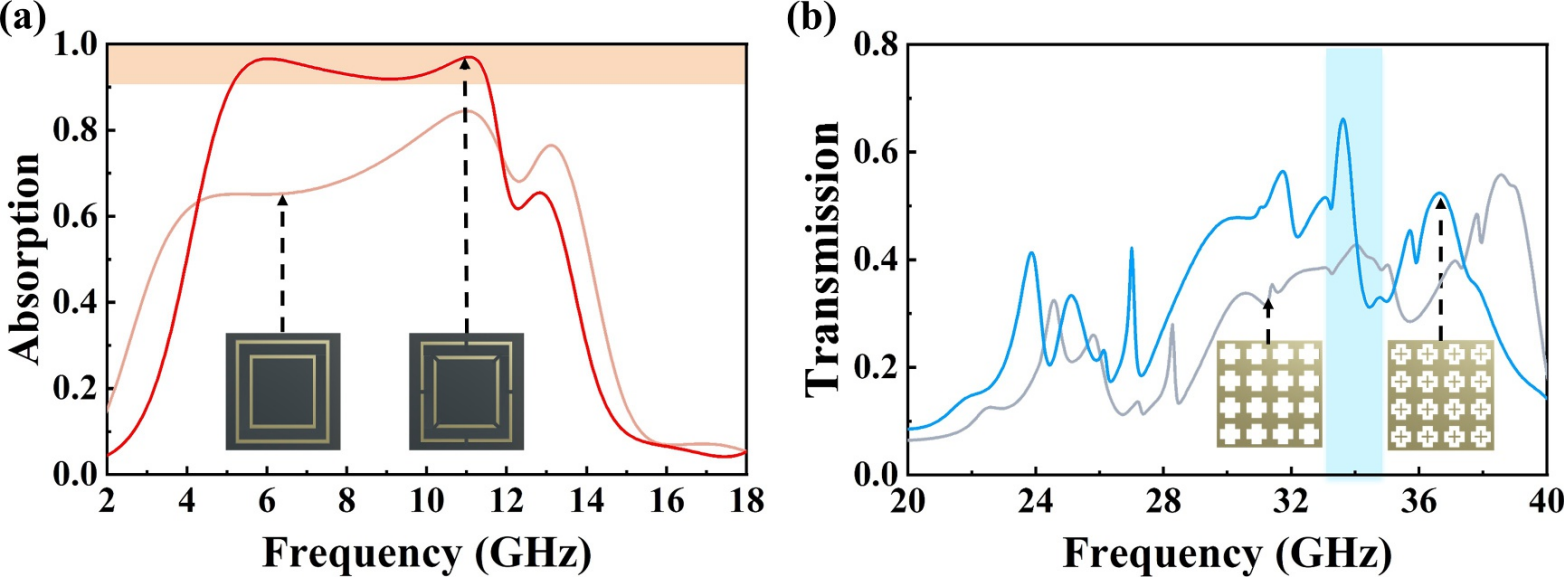
Simulated performance of the optimized metamaterial unit cell. (a) Absorption spectra comparing the top‐layer resonator with and without notches over 2–18 GHz. (b) Transmission spectra for the two candidate bottom‐layer FSS patterns over 20–40 GHz.

The optimization follows a stepwise co‐design approach focusing on the interplay between the top resonators and the bottom FSS. Initially, the bottom FSS was set as a perfect reflector, and the top‐layer pattern—starting from a solid double‐square‐ring—was evolved into a notched configuration as depicted in Figure 1a. Introducing notches to the double‐square‐ring resonator effectively broadened the absorption bandwidth within 2–18 GHz. After finalizing the top‐layer design, we proceeded to design the bottom FSS to create a transmission window in 20–40 GHz. Two candidate patterns were evaluated; their simulated transmission curves are summarized in the table of Figure 1. Between them, the pattern formed by embedding a smaller cross inside a larger cross exhibits a more concentrated transmission window within the target band.

### 2.2 Structural Architecture

Based on the above simulation and optimization results, the final three‐dimensional structure of the absorptive–transmissive integrated metamaterial is determined in this work, as shown in Figure 2a. This structure adopts a classic multilayer design, which consists, from top to bottom, of a patterned metallic resonant layer, a dielectric substrate layer, and a metallic mesh FSS layer. Among these, the finalized top resonant layer features a center‐symmetric notched double‐square‐ring structure (Figure 2b), where the notches on the inner and outer rings are offset by 45° to effectively excite multiple resonant modes for broadening the low‐frequency absorption bandwidth. The bottom FSS layer incorporates a composite pattern with an embedded cross‐shaped aperture (Figure 2e), designed to create a high‐transmission window around a specific high frequency (near 33.6 GHz). Furthermore, Figure 2c illustrates the overall periodic arrangement of the bottom FSS, whereas Figure 2d specifies the general geometric parameters of the metallic mesh.

**FIGURE 2:**
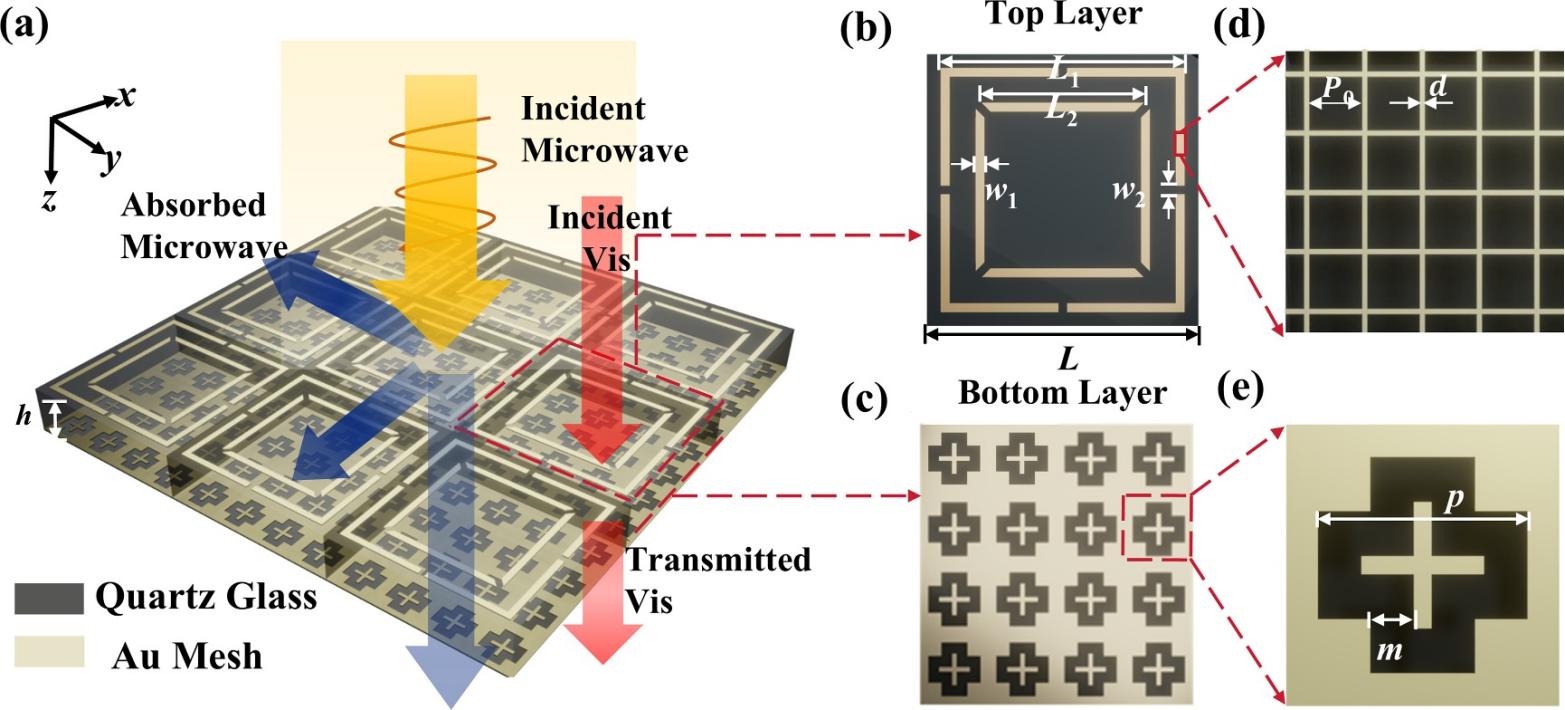
Architecture of the metamaterial window. (a) 3D schematic, (b) top resonator, (c) bottom FSS overview, (d) metal mesh parameters, and (e) bottom FSS unit cell.

The key geometric parameters of the resonant layer include the outer square side length $L_{1}$, the inner square side length $L_{2}$, the notch width $w_{1}$, and the ring linewidth $w_{2}$. For the FSS layer, the core parameters are the length p of the cross‐shaped aperture and its distance m from the central metallic strip. The dominant energy loss in the structure originates from the ohmic loss within the metallic mesh. The intensity of this loss is governed by the sheet resistance r, which directly dictates both the impedance matching condition and the dissipation characteristics of the absorber, as described by the following expression:

(1) $$r=\frac{1}{\sigma t}\cdot \frac{p_{0}}{d},$$

where σ represents the electrical conductivity of the metal, t is the metal thickness, $p_{0}$ denotes the period, and d is the linewidth. For given values of p and d, the sheet resistance r is inversely proportional to the thickness t. To achieve efficient absorption, the top resonant layer requires a relatively high sheet resistance to enhance energy dissipation, which is realized by using a smaller metal thickness ($t_{\text{top}}=120\;\text{nm}$). In contrast, the bottom FSS layer needs to approximate a perfect reflector at lower frequencies, necessitating a lower sheet resistance and, correspondingly, a greater metal thickness ($t_{\text{bottom}}=500\;\text{nm}$). Furthermore, the optical transmittance $T_{\text{opt}}$ of the device is predominantly determined by the filling factor of the metallic mesh, as given by the following equation:

(2) $$T_{\text{opt}}\approx {\left(1-\frac{d}{p_{0}}\right)}^{2}.$$

The metallic‐mesh parameters, including the mesh period and linewidth, require co‐optimization to simultaneously achieve high optical transmittance and the desired microwave impedance. In this work, gold metallic meshes are adopted as the conductive platform due to their high electrical conductivity and compatibility with standard microfabrication processes. The mesh linewidth is designed as d=5μm, whereas the mesh periods of the top resonant layer and bottom FSS layer are determined according to their different electromagnetic functions and fabrication constraints.

The effects of the resonant unit parameters ($L_{1},L_{2},w_{1},w_{2}$), FSS parameters (p,m), and dielectric spacer thickness h on the electromagnetic response were investigated, as presented in Supplementary Information Supporting Information S1: Figures S1–S3. The detailed metallic mesh design parameters and fabricated mesh morphologies are provided in Supporting Information S1: Figure S4. These optimizations establish a balanced trade‐off among absorption bandwidth, transmission performance, and optical transparency.

The final optimized structure consists of a unit‐cell period of L=15.6mm and a dielectric spacer thickness of h=4mm. The dimensions for the resonant layer are $L_{1}=14\;\text{mm}$, $L_{2}=10\;\text{mm}$, $w_{1}=w_{2}=0.5\;\text{mm}$, with a metal thickness of $t_{\text{top}}=120\;\text{nm}$; the dimensions for the FSS layer are p=3mm, m=0.5mm, with a metal thickness of $t_{\text{bottom}}=500\;\text{nm}$. Based on the electrical conductivity of gold ($\sigma =4.5\times 10^{7}S/m$), the calculated sheet resistances for the resonant layer and the FSS layer are 12Ω/▫ and 0.5Ω/▫, respectively.

Both mesh layers are fabricated from gold with a linewidth of 5μm. The top resonator is patterned via negative‐tone photolithography and lift‐off after magnetron sputtering of 120‑nm‑thick gold; the bottom FSS is patterned using positive‐tone photolithography and lift‐off after sputtering of 500‑nm‑thick gold on the opposite side of the 4‑mm‑thick8‑inch quartz wafer. The thick metal and narrow linewidths on the bottom layer can cause resist sidewall effects, which are mitigated by optimizing the photoresist thickness and exposure parameters. With these optimized parameters, the simulated performance of the final device is presented in Figure 3. The absorption spectrum (2–18 GHz) shows two distinct peaks at *f*_1_ and *f*_2_, whereas the transmission spectrum (20–40 GHz) features a passband centered at *f*_3_.

**FIGURE 3:**
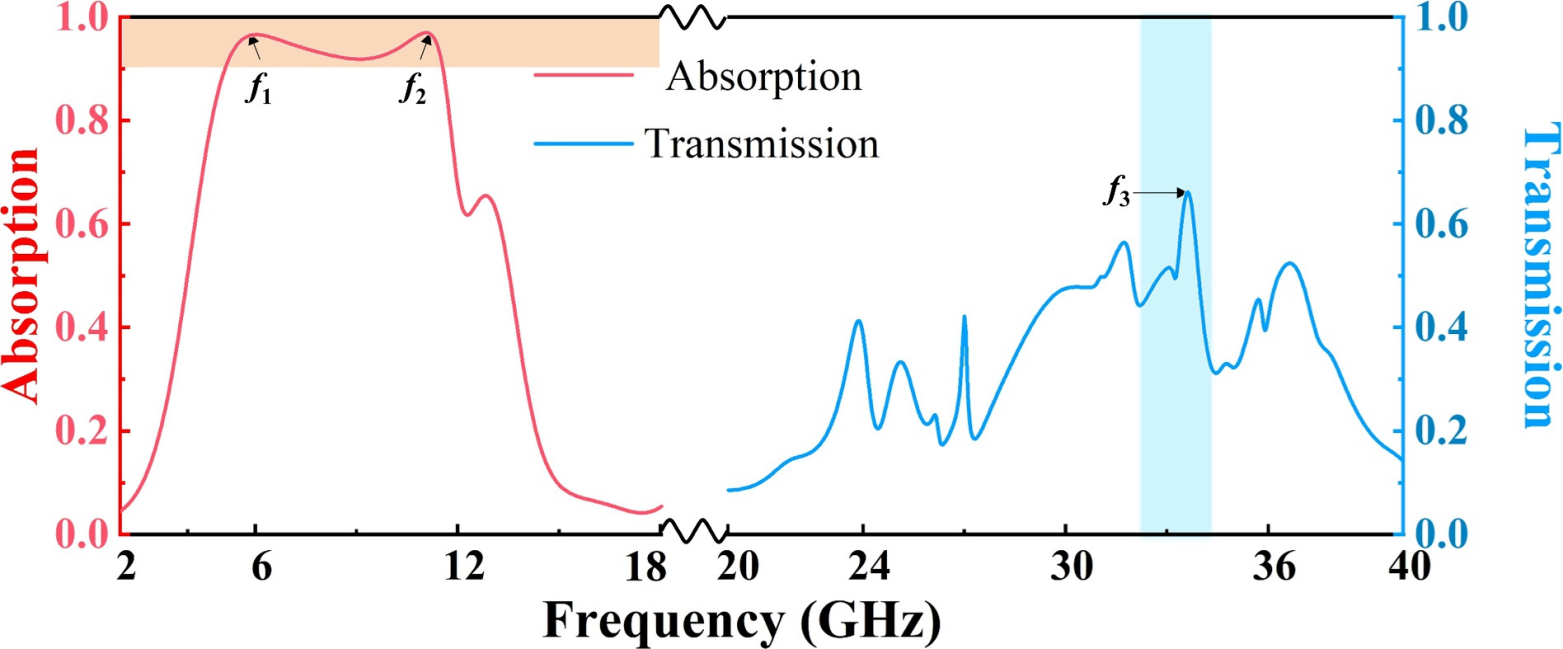
Simulated absorption (red, 2–18 GHz) and transmission (cyan, 20–40 GHz) spectra of the metadevice, indicating the absorption peaks *(f*_1_ *, f*_2_) and the transmission peak (*f*_3_).

### 2.3 Angular Response

In practical scenarios, electromagnetic waves are seldom at perfect normal incidence [44, 45]. To evaluate whether the angular stability of the device under oblique incidence meets application requirements, we analyzed its absorption characteristics under both transverse electric (TE) and transverse magnetic (TM) polarizations via full‐wave simulations. As shown in Figure 4a reveals that the angular robustness of the TE polarization exhibits distinct frequency selectivity. Specifically, around 11.9 GHz, the absorption rate remains above 90% even at an incident angle up to 60°. In contrast, at frequencies such as 5.8 GHz, the absorption performance degrades more rapidly with increasing angle. On the other hand, the TM polarization demonstrates more stable performance across the 5.8–11.9 GHz band (Figure 4b). Within an incident angle range of 0°–80°, the absorption curves exhibit gradual variations, maintaining notable stability especially between 0° and 75°. These results indicate that the structure offers good angular adaptability under TM polarization, whereas its angular characteristics for TE polarization are closely dependent on frequency.

**FIGURE 4:**
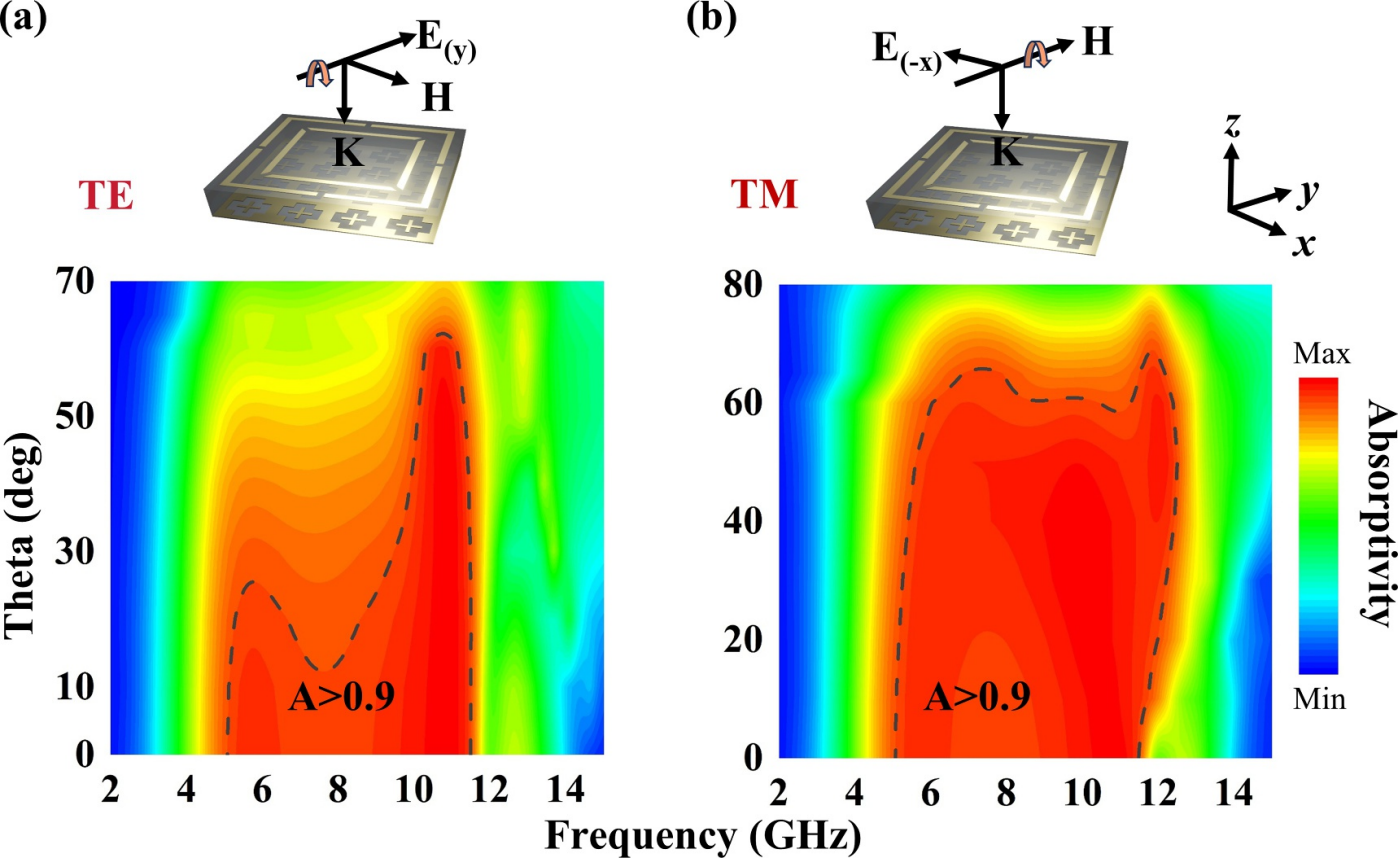
Simulated absorption under oblique incidence. (a) TE polarization. (b) TM polarization.

To clarify the origin of the frequency‐dependent angular degradation under TE polarization, the oblique‐incidence impedance matching behavior was analyzed. For the proposed resonant cavity structure, the absorption performance is governed by the matching between the effective input impedance and the wave impedance of free space. The wave impedances for TE and TM polarizations are expressed as $Z_{0}^{\text{TE}}=Z_{0}/\cos\;\theta$ and $Z_{0}^{\text{TM}}=Z_{0}\;\cos\;\theta .$ Although the TE impedance transformation alone does not directly determine the absorption variation, the effective surface impedance of the resonant multilayer structure becomes angle‐dependent due to the variation of resonant coupling and Fabry–Pérot phase accumulation under oblique incidence. For TE polarization, increasing the incident angle can enhance the deviation between the effective input impedance and the corresponding free‐space wave impedance, particularly at frequencies where the impedance matching margin is limited. Consequently, reflection increases and absorption decreases. At approximately 11.9 GHz, the structure maintains a strong impedance matching condition because of the optimized resonant coupling, resulting in stable absorption over a wide angular range. In contrast, near 5.8 GHz, where the impedance matching is weaker, the angular perturbation causes a more pronounced mismatch and therefore a faster reduction in absorption. For TM polarization, the opposite angular variation of the wave impedance introduces a compensation effect, leading to improved angular stability [46, 47].

## 3 Analysis of the Integrated Absorption and Transmission Mechanism

### 3.1 Loss Characteristics and Impedance Matching Mechanism

To elucidate the physical mechanism underlying the broadband and efficient absorption of the structure [20, 48], we conducted an analysis from two aspects: energy loss distribution and impedance matching characteristics. As shown in Figure 5a, the normalized power loss distribution across the functional layers indicates that over 90% of the incident energy is primarily concentrated and dissipated within the top metallic resonant structure, whereas the contributions from the dielectric spacer and the bottom layer are negligible. This clearly demonstrates that energy absorption is predominantly achieved by the top‐layer structure. Figure 5b shows that within the frequency range of 5–11.4 GHz, the equivalent normalized input impedance of the structure exhibits a real part close to 1 and an imaginary part approaching 0. This indicates that the structure achieves excellent impedance matching with free space across a broad bandwidth, thereby effectively suppressing reflection. Consequently, the outstanding absorption performance of this metamaterial window originates from the synergistic interaction between the efficient energy dissipation of the top resonant structure and the broadband impedance matching of the overall configuration.

**FIGURE 5:**
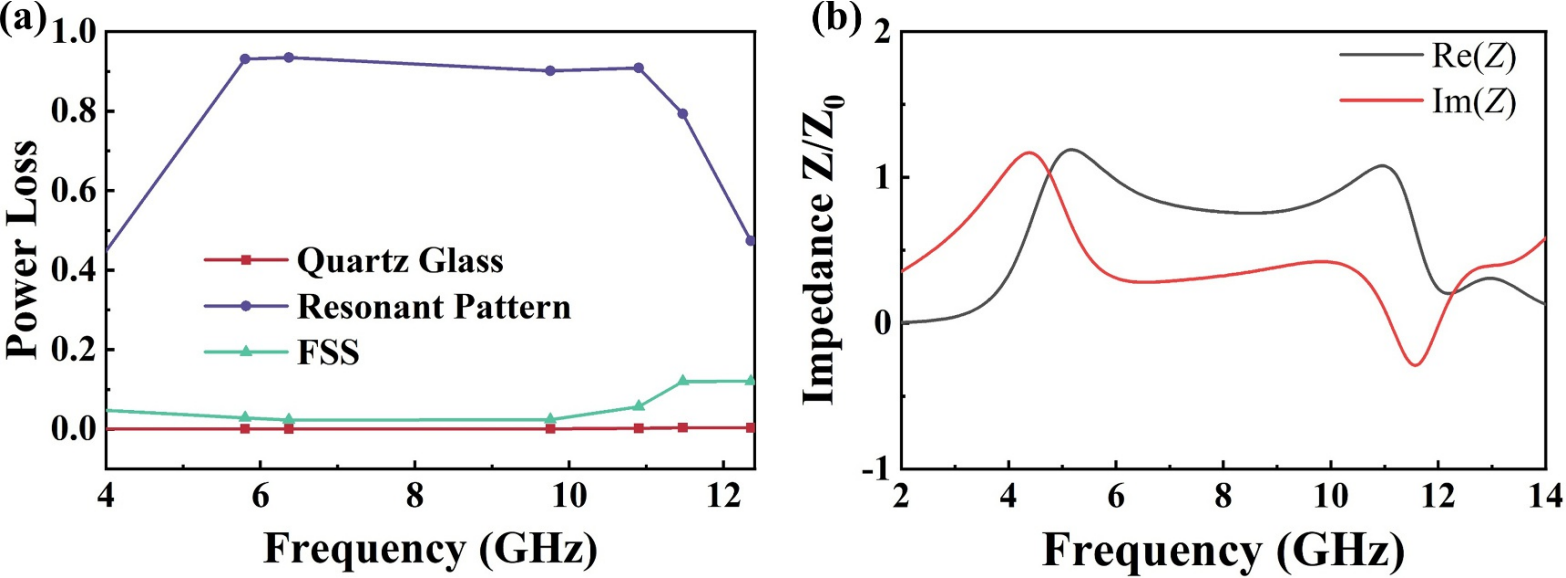
Analysis of power dissipation and impedance matching. (a) Layer‐resolved profile of the normalized power loss. (b) Real and imaginary parts of the normalized input impedance.

### 3.2 Multiple‐Reflection Interference Mechanism

Within the low‐frequency absorption band of 5–11.4 GHz, based on the aforementioned analysis of energy loss and impedance matching, the bottom FSS exhibits high reflectivity to incident waves and can therefore be approximated as a perfect electric conductor (PEC) ground plane. In this case, the bottom FSS satisfies the approximate boundary conditions $r_{23}\approx -1$ and $t_{23}\approx 0$. This property allows the entire structure, consisting of the top resonant layer, dielectric spacer, and bottom FSS, to be abstracted as a Fabry–Pérot resonant cavity. Electromagnetic waves undergo multiple reflections between the partially reflecting top layer and the PEC‐like bottom boundary, and the absorption characteristics can therefore be analyzed using the classical multiple‐reflection interference theory [49].

Figure 6a illustrates the multiple‐beam interference model. The oblique rays are only used to visualize the multiple‐reflection process, whereas all calculations and simulations are performed under normal incidence. Following the classical interference theory of thin‐film optics, the power reflection coefficient R of the structure can be expressed as the following equation:

(3) $$R={\left|{\tilde{r}}_{12}+\frac{{\tilde{t}}_{12}{\tilde{t}}_{21}{\tilde{r}}_{23}\;e^{i\;2\beta }}{1-{\tilde{r}}_{21}{\tilde{r}}_{23}\;e^{i\;2\beta }}\right|}^{2},$$

where $r_{ij}$ and $t_{ij}$ represent the Fresnel reflection and transmission coefficients, respectively, for the interface from medium i to medium j. The term $\beta =k_{0}nh$ represents the single‐pass phase delay through the dielectric spacer with thickness h and refractive index n, where $k_{0}$ is the free‐space wavenumber. Because the bottom FSS behaves as a PEC‐like reflector in this frequency range, the multiple‐reflection interference is mainly determined by the interaction between the top resonant layer and the bottom reflecting boundary. The absorption spectrum calculated from this interference model agrees well with the full‐wave simulation results, as shown in Figure 6c, confirming that destructive interference within the cavity is responsible for the broadband absorption.

**FIGURE 6:**
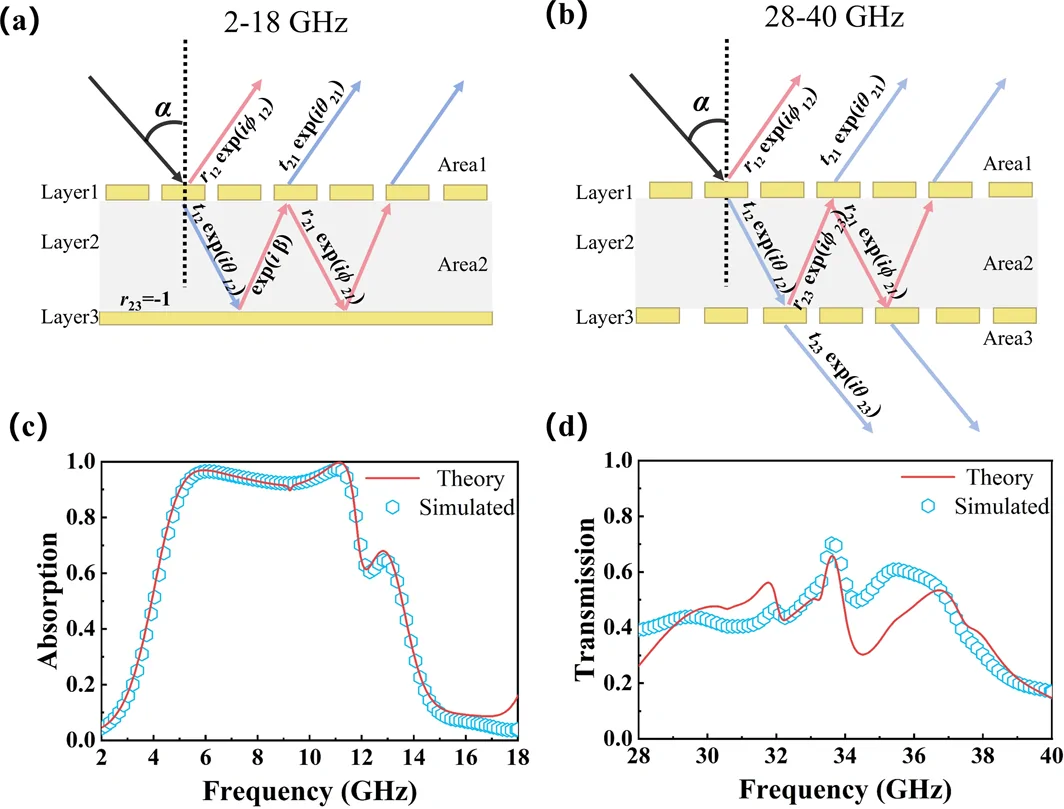
Analytical interference model and its numerical validation. Schematic illustrations of the proposed multiple‐beam interference models: (a) absorption and (b) transmission. Comparison between FEM simulations (blue circles) and analytical calculations (red lines): (c) absorption spectrum and (d) transmission spectrum.

The above PEC approximation is only valid in the low‐frequency absorption region. Within the transmission window of 28–40 GHz, the electromagnetic response of the bottom FSS changes significantly due to the resonance of the cross‐shaped composite unit. Therefore, the bottom FSS no longer behaves as an ideal reflector, but instead acts as a frequency‐selective transmitting layer. As illustrated in Figure 6b, the bottom FSS is introduced into the Fabry–Pérot model as a partially transmitting interface. Unlike the PEC case with fixed coefficients, the reflection and transmission coefficients of the FSS in this frequency range are frequency‐dependent complex parameters and must be obtained from its actual electromagnetic response.

To extract these parameters, the bottom FSS is modeled as a reciprocal two‐port network. A stand‐alone infinite periodic unit cell is simulated using Floquet‐port excitation, and the reference planes are de‐embedded from the FSS surfaces [50]. The obtained scattering parameters $S_{11}^{\text{FSS}}$ and $S_{21}^{\text{FSS}}$ describe the reflection and transmission characteristics of the FSS layer. Considering reciprocity and structural symmetry, $S_{12}^{\text{FSS}}=S_{21}^{\text{FSS}}$ and $S_{22}^{\text{FSS}}=S_{11}^{\text{FSS}}$. The S parameters are subsequently converted into the ABCD transmission matrix using the standard network transformation:

(4) $$\left[\begin{array}{cc} A & B\\ C & D \end{array}\right]=\left[\begin{array}{cc} \frac{\left(1+S_{11}\right)\left(1-S_{22}\right)+S_{12}S_{21}}{2S_{21}} & Z_{0}\frac{\left(1+S_{11}\right)\left(1+S_{22}\right)-S_{12}S_{21}}{2S_{21}}\\ \frac{1}{Z_{0}}\frac{\left(1-S_{11}\right)\left(1-S_{22}\right)-S_{12}S_{21}}{2S_{21}} & \frac{\left(1-S_{11}\right)\left(1+S_{22}\right)+S_{12}S_{21}}{2S_{21}} \end{array}\right].$$

For the symmetric FSS structure, $S_{11}=S_{22}$, and the above equation is defined as Equation (4). The field reflection and transmission coefficients of the bottom FSS are then retrieved from the ABCD matrix as follows:

(5) $$r_{23}=\frac{A+B/Z_{0}-CZ_{0}-D}{A+B/Z_{0}+CZ_{0}+D},$$

(6) $$t_{23}=\frac{2}{A+B/Z_{0}+CZ_{0}+D}.$$

This retrieval process preserves both amplitude and phase information, allowing the frequency‐dependent response of the FSS to be directly incorporated into the multiple‐reflection interference model. The total transmission coefficient of the complete structure is therefore expressed as follows:

(7) $$T_{\text{total}}=\frac{t_{12}t_{23}e^{-j\beta }}{1-r_{21}r_{23}e^{-j2\beta }}.$$

The analytical transmission spectrum calculated from Equation (7) agrees well with the full‐wave simulation results in the 28–40 GHz band, as shown in Figure 6d, validating the S‐parameter retrieval method and the modified interference model.

Furthermore, the high‐frequency transmission model is consistent with the low‐frequency PEC model. When the retrieved coefficients approach the PEC condition ($r_{23}\approx -1$, $t_{23}\approx 0$), Equation (7) reduces to the original multiple‐reflection interference model. Therefore, the absorption and transmission characteristics over the entire operating band can be explained by a unified Fabry–Pérot interference mechanism. The bottom FSS provides different frequency‐dependent boundary conditions: it behaves as a PEC‐like reflector in the absorption band, producing destructive interference and strong absorption, whereas it functions as a resonant transmitting layer in the transmission band, determining the formation of the passband.

### 3.3 Field and Current Distributions at Resonance

To clarify the physical mechanisms underlying the “absorption” and “transmission” modes, a systematic analysis was conducted at three characteristic frequencies [51]. The overall performance, shown in Figure 3, features broadband absorption in the 5–11.4 GHz band and a selective transmission window in the 20–40 GHz band. Accordingly, two resonant frequencies within the absorption band (*f*_1_ = 5.8 GHz and *f*_2_ = 10.9 GHz) and the transmission peak frequency (*f*_3_ = 33.6 GHz) were selected for detailed examination. The analysis focuses on their electric‐field distributions, power‐loss densities, and surface‐current patterns.

At the absorption frequencies *f*_1_ and *f*_2_, the electromagnetic field responses exhibit a high degree of consistency, and their physical picture can be uniformly described as a process of “LSPR–ohmic loss conversion.” The electric field (Ez component) is strongly concentrated at the gaps and edges of the top metallic mesh (Figure 7a), indicating the excitation of intense LSPR, which effectively traps and localizes the incident electromagnetic energy. The corresponding normalized power‐loss distribution (Figure 7b) shows that the regions of high loss coincide precisely with the aforementioned electric‐field concentration zones, with the vast majority (> 90%) of the loss occurring in the top metallic mesh. The surface current distribution (Figure 7c) further confirms that strong closed‐loop vortex currents are induced in the top mesh at these frequencies. This current efficiently converts the localized electromagnetic energy into thermal energy via Joule heating (*P_*loss = *I^2^R*), which aligns with the loss mechanism of classical metamaterial absorbers [52, 53, 54]. Therefore, the essence of the low‐frequency broadband absorption is that the top resonant structure localizes energy through LSPR and completes the electromagnetic‐to‐thermal energy conversion via ohmic loss.

**FIGURE 7:**
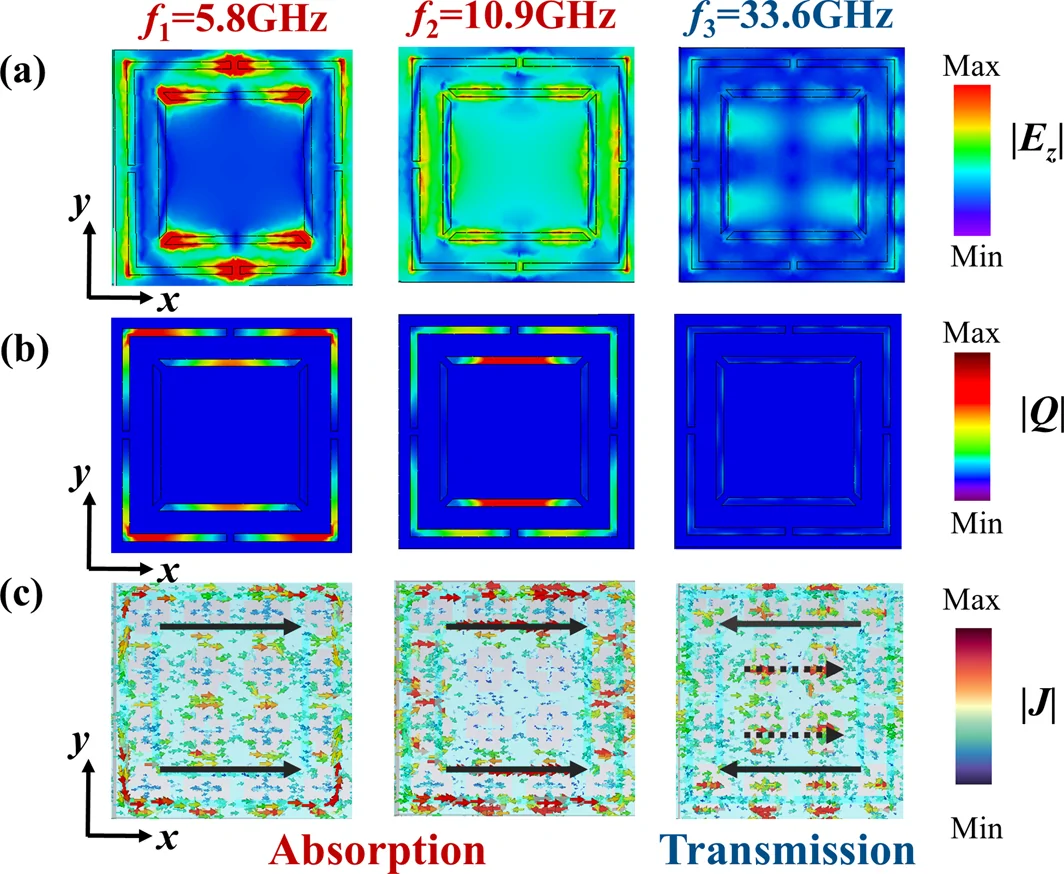
Near‐field characteristics at representative frequencies. (a) Spatial distributions of the out‐of‐plane electric field ($E_{z}$). (b) Surface power loss density distributions. (c) Surface current distributions of the top resonant layer and bottom FSS. Solid arrows indicate the current direction on the top resonant layer, whereas dashed arrows denote the current direction on the cross‐shaped FSS elements. The field distributions in (a) and (b) are evaluated at 5.8, 10.9, and 33.6 GHz, corresponding to the representative absorption and transmission states.

At the transmission frequency $f_{3}$, the field distributions exhibit distinctly different characteristics. Compared with the absorption states, the transmission mode can be understood from the perspective of coherent scattering manipulation based on the Huygens equivalent principle [55, 56]. First, the electric field uniformly penetrates the entire structure with relatively low amplitude (Figure 7a), whereas the power‐loss distribution remains at an extremely low level overall (Figure 7b). This observation, from an energy perspective, confirms that the incident wave experiences minimal perturbation. The most critical evidence comes from the surface current analysis (Figure 7c). Compared with the absorption state, the surface current intensity on the top resonant structure is significantly reduced, accompanied by a reversed current direction. Meanwhile, the cross‐shaped elements of the bottom FSS exhibit enhanced symmetric current excitation. More importantly, the induced currents on the top resonator and bottom FSS are oriented in opposite directions, forming an antiphase coupled current configuration. This antiphase coupled current response indicates that the top resonator and bottom FSS act as coupled electromagnetic dipole sources under external excitation. According to the Huygens equivalent principle, the interaction between these induced electric and magnetic dipole responses governs the phase and amplitude of the far‐field scattering. Specifically, the backward‐scattered fields generated by the top resonator and bottom FSS experience destructive interference due to their opposite phase relationship, thereby suppressing the reflected wave. Meanwhile, the forward‐scattered components remain coherently combined with the incident field, enabling efficient transmission through the structure [57]. Therefore, the antiphase synergistic current mode at $f_{3}$ represents a coherent scattering‐control process rather than an energy‐dissipative resonance, allowing the structure to achieve suppressed reflection and high‐efficiency transmission around $f_{3}$.

## 4 Experimental Validation

To validate the practical performance of the designed metamaterial window, we fabricated a sample and systematically characterized its optical and microwave properties. The sample was fabricated on a 4‐mm‐thick, 8‐inch quartz‐glass substrate using standard photolithography and magnetron sputtering processes.

The detailed metallic‐mesh parameters are illustrated in Supporting Information S1: Figure S4. Specifically, both the top resonant mesh and bottom FSS mesh employ a linewidth of d=5μm, whereas different mesh periods and metal thicknesses are adopted according to their electromagnetic functions. The fabricated top resonant mesh has a period of $p_{0\_\text{top}}=300\;\mu m$ with $t_{\text{top}}=120\;\text{nm}$, whereas the bottom FSS mesh has a period of $p_{0\_\text{bottom}}=55\;\mu m$ with $t_{\text{bottom}}=500\;\text{nm}$.

The specific fabrication flow was as follows: first, the top resonant layer (120‐nm‐thick gold) was patterned on the front side of the substrate using negative‐tone photolithography and a lift‐off process. Subsequently, the bottom frequency‐selective surface (500‐nm‐thick gold) was fabricated on the back side using positive‐tone photolithography to accommodate the process variations introduced by the thick substrate. During fabrication, a systematic optimization of the photoresist thickness and exposure parameters was conducted to effectively control the pattern transfer quality, mitigating the issue of “resist sidewalls” that easily form after development due to the thick metal and fine linewidth of the bottom layer. This optimization ultimately yielded a sample with intact structures, as shown in Figure 8a.

**FIGURE 8:**
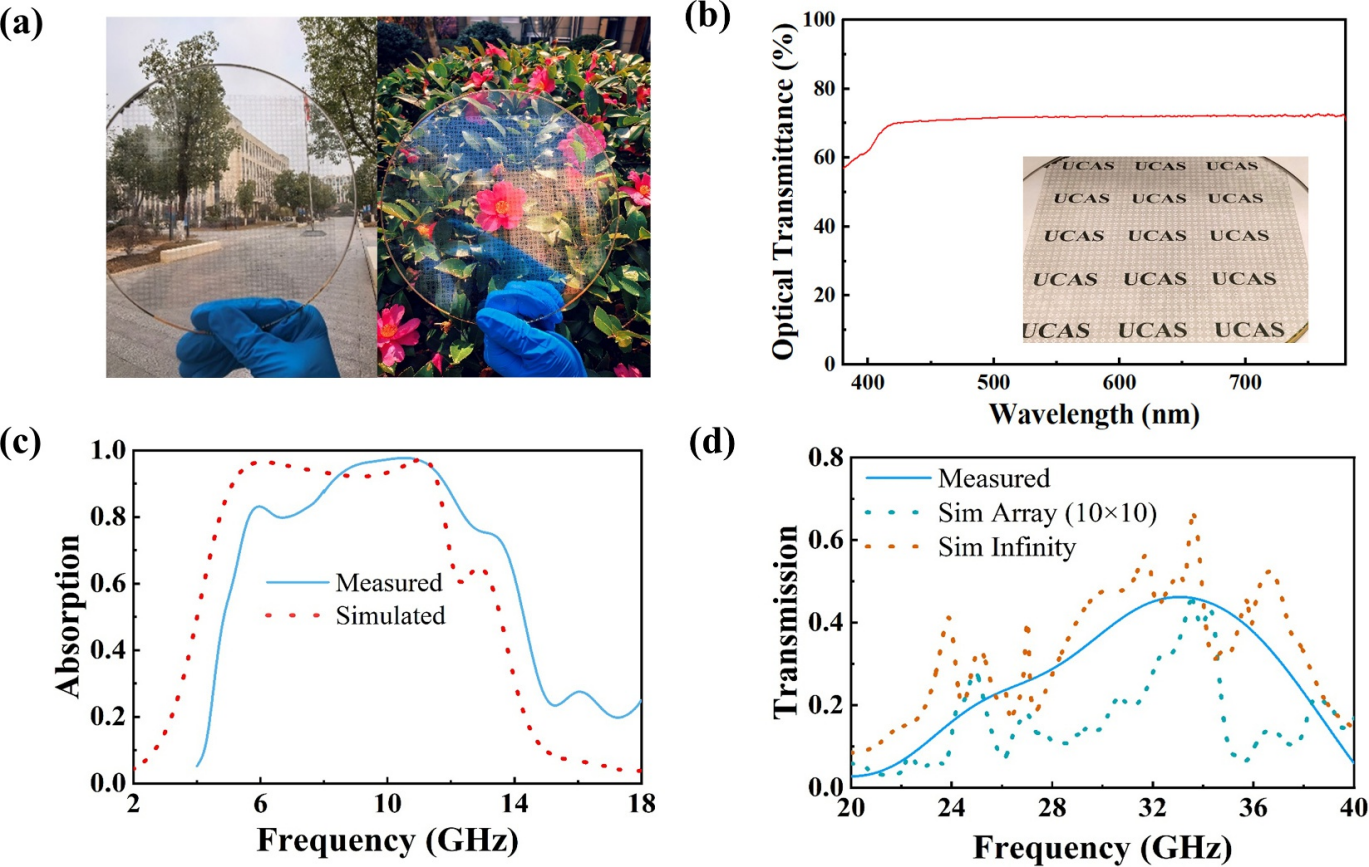
Fabrication and experimental characterization. (a) Photograph of the fabricated metamaterial window prototype. (b) Measured optical transmission spectrum. (c) Absorption spectra: comparison between infinite unit‐cell simulation and measurement. (d) Transmission spectra: comparison among infinite periodic simulation, finite 10 × 10 array simulation, and experimental measurement.

The optical transmission spectrum was measured using a UV–vis spectrophotometer (Shimadzu UV‐3600) equipped with a D65 illumination source over the wavelength range of 380–780 nm. The average visible transmittance was calculated by taking the arithmetic average of the measured spectral transmittance values within this wavelength range using Origin software. As shown in Figure 8b, the fabricated sample exhibits good optical transparency, with an average transmittance of 68.5% and a peak transmittance of 72.2% at 550 nm. The reduction in optical transmission is mainly attributed to two factors: the slightly increased metallic filling factor caused by fabrication deviations and Fresnel reflection losses at the air–quartz interfaces [42, 58].

The selection of the 4‐mm‐thick quartz substrate represents a compromise among mechanical robustness, structural weight, and electromagnetic/optical performance. As discussed in Supporting Information S1: Figure S3a,b, the dielectric spacer thickness was systematically optimized by comparing quartz substrates with thicknesses ranging from 2.5 to 5 mm. The absorption and transmission responses were evaluated separately to identify the optimal thickness. The substrate thickness determines the phase accumulation within the dielectric spacer and consequently influences both the Fabry–Pérot interference condition for broadband absorption and the transmission response near 34 GHz. The selected thickness of 4 mm provides a favorable balance between maintaining the 5–11.4 GHz broadband absorption performance and preserving the high‐frequency transmission characteristic. Although a thicker quartz substrate inevitably introduces additional weight and increases Fresnel reflection at the air–quartz interfaces, the rigid quartz platform offers superior thermal stability, environmental durability, and power‐handling capability compared with flexible polymer‐based substrates. Therefore, the selected substrate thickness is considered suitable for electromagnetic window applications in harsh environments. Further reduction in weight could be achieved through lightweight rigid substrates, dielectric structural optimization, or antireflection coatings.

Microwave measurement results confirm the core electromagnetic functions of the device (Figure 8c,d). Together with the optical characterization, these results experimentally verify the simultaneous realization of optical transparency, broadband microwave absorption, and frequency‐selective transmission within a single passive electromagnetic window. Compared with previously reported multifunctional transparent electromagnetic devices (Supporting Information S1: Table S1) [10, 13, 14, 28, 29], which typically emphasize either absorption or transmission functionality, the proposed design provides an integrated platform combining these complementary electromagnetic responses. The detailed experimental configurations are provided in Supporting Information S1: Figure S5, including the schematic diagrams and photographs of both measurement setups. The reflection and transmission coefficients were obtained from the measured scattering parameters, and the absorption and transmission spectra were calculated according to $A=1-|S_{11}{|}^{2}-|S_{21}{|}^{2}$ and $T=|S_{21}{|}^{2}$, respectively. Within the 5–11.4 GHz absorption band, the measured average absorption is 85%, which aligns well with the full‐wave simulation result (92%), indicating good realizability of the broadband absorption design. Near the transmission window around 34 GHz, the measured peak transmittance is approximately 45%. As shown in Figure 8d, the infinite periodic unit‐cell simulation predicts a higher transmission peak of approximately 70%, whereas the finite 10 × 10 array simulation reduces the peak transmission to approximately 45%, which agrees well with the experimental result. This comparison demonstrates that finite‐size effects, including edge diffraction and radiation leakage, are the dominant factors responsible for the reduction of the transmission peak compared with the ideal periodic assumption. This difference is more pronounced in the high‐frequency transmission band because the transmission state relies on precise phase coupling between the top resonant layer and the bottom FSS, whereas the low‐frequency absorption response is mainly determined by broadband impedance matching and dissipative loss.

Although the finite‐array simulation reproduces the transmission peak amplitude, the measured transmission spectrum exhibits a broader resonance profile. This difference is mainly attributed to fabrication‐induced structural variations, including linewidth fluctuation, dimensional deviation, and local defects of the metallic mesh. To further evaluate the influence of fabrication‐related electrical variations, the effective sheet resistance of the bottom FSS was parametrically analyzed, and the results are provided in Supporting Information S1: Figure S6. Increasing the effective sheet resistance mainly reduces the transmission amplitude, whereas the resonance frequency remains nearly unchanged. This indicates that fabrication‐induced electrical variations primarily affect the transmission efficiency and spectral broadening rather than determining the resonance position [59, 60, 61]. Therefore, the deviation between simulation and measurement can be attributed to two independent effects: the reduction of the transmission peak from approximately 70%–45% is mainly caused by the finite size of the fabricated 10 × 10 array, whereas the broader experimental resonance profile is related to fabrication‐induced structural and electrical nonuniformities.

## 5 Conclusion

This paper presents the successful development of an absorptive–transmissive integrated metamaterial window based on quartz‐glass and micrometer‐scale metallic meshes. The device achieves an average broadband absorption of 85% in the 5–11.4 GHz band, forms a selective transmission window around 34 GHz, and maintains an average visible‐light transmittance of 68.5%. Through the co‐design of a rigid substrate and low‐filling‐factor fine meshes, a favorable balance is struck among optical transparency, specific electromagnetic functions, and environmental robustness. Simulations and experiments validate its performance. Physical mechanism analysis reveals that absorption originates from resonant loss, whereas transmission is governed by coupling interference. Deviations in high‐frequency transmission are primarily influenced by fabrication precision and finite‐size effects, pointing the way for subsequent optimization. This work provides a new design paradigm based on a reliable rigid platform for high‐performance integrated optical‐electromagnetic windows intended for harsh environments.

## Author Contributions

**Chaoyue Li:** writing – original draft, methodology, formal analysis, software, validation, data curation. **Chenyang Cui:** writing – review and editing, methodology, formal analysis, software, validation. **Wangqi Mao:** investigation, writing – review and editing, validation. **Pingping Min:** writing – review and editing, formal analysis, supervision, validation. **Tingting Yu:** investigation, data curation, software. **Yang Zhang:** investigation, data curation. **Yaqiang Zhang:** investigation, conceptualization, data curation. **Chi Zhang:** writing – review and editing, conceptualization, funding acquisition, visualization, supervision, formal analysis, software, project administration. **Hongxing Dong:** project administration, resources, supervision. **Long Zhang:** investigation, resources, supervision, project administration.

## Funding

This work was supported in part by the National Key R&D Program of China (Grant 2025YFF0520501) and by the National Natural Science Foundation of China (Grants 12404487 and 52502091).

## Supporting information


Supporting Information S1

## Data Availability

The data that support the findings of this study are available from the corresponding author upon reasonable request.
