# Supplementary material for: An Optically Transparent Metamaterial Window Based on Metal Meshes With Integrated Absorption and Transmission
Source: Nanophotonics. 2026 Aug 23;15(16):e70265. doi: 10.1002/nap2.70265 (PMC13672147; doi:10.1002/nap2.70265)
Supplement: Supplementary file 1 [file NAP2-15-e70265-s001.docx]

Supporting Information

**An Optically Transparent Metamaterial Window Based on Metal Meshes with Integrated Absorption and Transmission**

*Chaoyue Li ^1, †^, Chenyang Cui^2, 3, †^, Wangqi Mao ^4^, Pingping Min ^3, *^, Tingting Yu ^5^, Yang Zhang ^5^, Yaqiang Zhang ^6, *^, Chi Zhang ^1, *^, Hongxing Dong ^1, 4^, Long Zhang ^1, 4^*

^1^ Hangzhou Institute for Advanced Study, University of Chinese Academy of Sciences, Hangzhou, China
^2^ Harbin Institute of Technology, Harbin, China
^3^ Zhengzhou Advanced Research Institute of Harbin Institute of Technology, Zhengzhou, China

^4^ Key Laboratory of Materials for High-Power Laser, Shanghai Institute of Optics and Fine Mechanics, Chinese Academy of Sciences, Shanghai, China
^5^ Hangzhou Hengying Technology Co., Ltd, Hangzhou, China

^6^ Hangzhou Institute of Optics and Fine Mechanics, Hangzhou, China

^†^ Chaoyue Li and Chenyang Cui contributed equally to this work.

^*^ *Corresponding author. E-mail addresses: minpp@hit.edu.cn(Pingping Min), zhangyaqiang@siom.ac.cn(Yaqiang Zhang), chizhang@ucas.ac.cn(Chi Zhang)*

S1

**Fig. S1.** Schematic diagrams of parameter optimization: (a) Absorption spectra and (b) transmission spectra at different $L_{1}$; (c) Absorption spectra and (d) transmission spectra at different $L_{2}$.

A parametric sweep of the outer and inner square ring side lengths $L_{1}$ and $L_{2}$ of the top resonator was performed. As $L_{1}$ increases from 12 mm to 15 mm, the absorption peak near 5 GHz redshifts with reduced amplitude, while the peak near 11 GHz blueshifts with enhanced amplitude. The overall low‑frequency absorption bandwidth reaches its maximum at $L_{1}=14\text{ }\text{mm}$. The 34 GHz transmission peak first increases and then decreases with increasing $L_{1}$. As $L_{2}$ increases from 8 mm to 12 mm, the low‑frequency absorption bandwidth first broadens and then narrows, achieving the widest bandwidth at $L_{2}=10\text{ }\text{mm}$. The 34 GHz transmission peak initially increases and then saturates, reaching its maximum at $L_{2}=10\text{ }\text{mm}$. Based on these results, $L_{1}=14\text{ }\text{mm}$ and $L_{2}=10\text{ }\text{mm}$ were selected as the optimal parameters.

S2:

**Fig. S2.** Schematic diagrams of parameter optimization: (a) Absorption spectra and (b) transmission spectra at different $p$; (c) Absorption spectra and (d) transmission spectra at different $m$**.**

After determining the top‑layer parameters, a parametric sweep of the cross‑aperture width $p$ and the slit width $m$ of the bottom FSS was conducted. As $p$ increases from 2 mm to 3.5 mm, the low‑frequency absorption peaks redshift and the absorption bandwidth gradually narrows. The high‑frequency transmission peak is significantly affected, reaching its maximum at $p=3\text{ }\text{mm}$. As $m$ increases from 0.25 mm to 0.625 mm, the low‑frequency absorption peaks redshift, and the bandwidth first broadens and then narrows, with the widest bandwidth observed at $m=0.5\text{ }\text{mm}$. The high‑frequency transmission peak initially increases and then saturates, also reaching its optimum at $m=0.5\text{ }\text{mm}$. Accordingly, $p=3\text{ }\text{mm}$ and $m=0.5\text{ }\text{mm}$ were selected as the optimal bottom‑FSS parameters.

S3：

**Fig. S3.** Influence of quartz substrate thickness on the electromagnetic response. (a) Simulated absorption spectra of the proposed structure with quartz substrate thicknesses ranging from 2.5 mm to 5 mm. (b) Simulated transmission spectra corresponding to different quartz substrate thicknesses. The 4-mm-thick quartz substrate provides an optimized trade-off between broadband absorption in the 5–11.4 GHz band and high-frequency transmission performance around 34 GHz.

S4: The metal thicknesses were initially determined according to the required sheet resistance and electromagnetic functions, after which the mesh periods were optimized by balancing optical transparency, impedance matching, and fabrication resolution. Considering the electrical conductivity of gold and the required microwave sheet resistance, the metal thicknesses of the top resonant layer and bottom FSS layer were determined first. Subsequently, the mesh periods were optimized by balancing electromagnetic performance, optical filling ratio, and the resolution limitation of the photolithography process. The detailed design parameters and fabricated mesh morphologies are provided in Fig. S4. Here, $p_{0}$denotes the physical mesh period of the fabricated metallic grid, whereas $p$and $m$represent the electromagnetic unit-cell parameters used for FSS simulation.

**Fig. S4.** Schematic illustration and optical microscopy characterization of the fabricated metallic meshes.

(a) Schematic illustration of the top resonant mesh and bottom FSS mesh structures. Both metallic meshes employ a linewidth of $d=5\text{ }\mu m$, while different metal thicknesses are adopted according to their distinct electromagnetic functions. The top resonant layer has a thickness of $t_{\mathrm{top}}=120\text{ }nm$, whereas the bottom FSS layer has a thickness of $t_{\mathrm{bottom}}=500\text{ }nm$. Based on the conductivity of gold ($\sigma=4.5\times{10}^{7}\text{ }S/m$), the required sheet resistance was first determined using Eq. (1), and the mesh periods were subsequently optimized by considering the trade-off between microwave impedance matching, optical transparency, and fabrication resolution.

(b) Optical microscopy image of the fabricated top resonant metallic mesh. The fabricated structure exhibits a mesh period of $p_{0\_top}=300\text{ }\mu m$, linewidth of $d=5\text{ }\mu m$, and metal thickness of $t_{\mathrm{top}}=120\text{ }nm$.

(c) Optical microscopy image of the fabricated bottom FSS metallic mesh. The fabricated structure exhibits a mesh period of $p_{0\_bottom}=55\text{ }\mu m$, linewidth of $d=5\text{ }\mu m$, and metal thickness of $t_{\mathrm{bottom}}=500\text{ }nm$.

S5：

**Fig. S5.** Experimental setups for microwave characterization of the fabricated metamaterial window. (a) Schematic illustration of the free-space measurement method for obtaining reflection and transmission coefficients. (b) Photograph of the free-space measurement setup. (c) Schematic illustration of the arch measurement method for transmission characterization. (d) Photograph of the arch measurement setup.

The free‑space method (panels (a) and (b)) employs a vector network analyzer (Keysight N5227B) and two horn antennas placed 1.5 m apart on opposite sides of the sample, providing quasi‑plane‑wave illumination at normal incidence. Conical absorbers suppress stray reflections, and time‑domain gating eliminates edge diffraction. The absorption and transmission are obtained from the measured S‑parameters as $A=1-\mid S_{11}\mid^{2}-\mid S_{21}\mid^{2}$ and $T=\mid S_{21}\mid^{2}$ The arch method (panels (c) and (d)) uses two antennas symmetrically mounted on a curved rail. A reference metal plate is measured first for calibration; the sample is then tested under identical angle and polarization conditions. The system uncertainty is approximately ±0.8 dB.

S6:

The sheet resistance of a metallic mesh is determined by $R_{s}\propto1/(\sigma\cdot t\cdot\text{duty}\text{ }\text{ratio})$, where $\sigma$ is the effective conductivity, $t$ is the metal thickness, and the duty ratio is the line width divided by the period. Fabrication imperfections such as over‑etching reduce $t$, lateral etching narrows the line width (lower duty ratio), and surface roughness degrades $\sigma$. All these increase $R_{s}$. To evaluate their impact, we swept $r_{1}$ and $r_{2}$.

**Fig. S6.** Parametric optimization of the sheet resistances $r_{1}$ and $r_{2}$. (a) Absorption and (b) transmission spectra for different top‑layer sheet resistances $r_{1}$. (c) Absorption and (d) transmission spectra for different bottom‑FSS sheet resistances $r_{2}$.

As $r_{1}$ increases from $5\text{ }\Omega/\square$ to $15\text{ }\Omega/\square$, the 5 GHz absorption peak decreases, the 11 GHz peak rises slightly, and the 34 GHz transmission remains nearly unchanged. As $r_{2}$ increases from $0.5\Omega/\square$ to $1.2\text{ }\Omega/\square$, the low‑frequency absorption stays stable, but the 34 GHz transmission peak drops from ~80% to ~55%. This confirms that $r_{2}$ is the dominant parameter for transmission. In the fabricated device, the combination of over‑etching, lateral etching, and surface roughness caused $r_{2}$ to exceed the scanned range, consistent with the measured 45% transmission. Together with the finite‑array effect (~2 dB reduction), these process‑induced increases in $r_{2}$ provide a quantitative explanation for the observed performance. For optimal design, $r_{1}=9$-$11\text{ }\Omega/\square$ and $r_{2}=0.5-0.6\text{ }\Omega/\square$ are recommended. The chosen $r_{2}=0.5\text{ }\Omega/\square$ balances transmission efficiency and fabrication feasibility.

Table S1. Comparison of representative multifunctional transparent electromagnetic windows and absorptive–transmissive metamaterial devices.

| **Substrate** | **Optical Transmittance** | **Absorption Band** | **Transmission Window** | **Ref.** |
| --- | --- | --- | --- | --- |
| Quartz glass | 65.5% | 3.6–15.8 GHz | — | [10] |
| Polycarbonate (PC) | 52.1% | — | 4.6–5.8 GHz | [14] |
| BOROFLOAT®33 glass | 86% @550 nm | — | 25–35 GHz | [15] |
| 3D-printed photopolymer resin | — | 12–18 GHz | 3.5–7 GHz | [31] |
| Photosensitive resin | — | 3.33–18 GHz | 8.79–10.4 GHz | [32] |
| **Quartz glass** | **68.5%** | **5–11.4 GHz** | **~34 GHz** | **This work** |

**Note:** "—" indicates that the corresponding performance was **not reported** in the referenced work.
